# EEG-Based Driver Fatigue Monitoring within a Human–Ship–Environment System: Implications for Ship Braking Safety

**DOI:** 10.3390/s23104644

**Published:** 2023-05-10

**Authors:** Bin Ren, Wanli Guan, Qinyu Zhou, Zilin Wang

**Affiliations:** 1 Shanghai Key Laboratory of Intelligent Manufacturing and Robotics, School of Mechatronic Engineering and Automation, Shanghai University, Shanghai 200444, Chinazqy1101@shu.edu.cn (Q.Z.); wzlshun@shu.edu.cn (Z.W.); 2 Zhejiang Key Laboratory of Robotics and Intelligent Manufacturing Equipment Technology, Ningbo Institute of Materials Technology & Engineering, Chinese Academy of Sciences, Ningbo 315201, China

**Keywords:** navigational safety, driver fatigue, human–ship–environment monitoring system, navigational safety, Stroop task experiment, centroid frequency, power spectral entropy, ridge regression model

## Abstract

To address the uncontrollable risks associated with the overreliance on ship operators’ driving in current ship safety braking methods, this study aims to reduce the impact of operator fatigue on navigation safety. Firstly, this study established a human–ship–environment monitoring system with functional and technical architecture, emphasizing the investigation of a ship braking model that integrates brain fatigue monitoring using electroencephalography (EEG) to reduce braking safety risks during navigation. Subsequently, the Stroop task experiment was employed to induce fatigue responses in drivers. By utilizing principal component analysis (PCA) to reduce dimensionality across multiple channels of the data acquisition device, this study extracted centroid frequency (CF) and power spectral entropy (PSE) features from channels 7 and 10. Additionally, a correlation analysis was conducted between these features and the Fatigue Severity Scale (FSS), a five-point scale for assessing fatigue severity in the subjects. This study established a model for scoring driver fatigue levels by selecting the three features with the highest correlation and utilizing ridge regression. The human–ship–environment monitoring system and fatigue prediction model proposed in this study, combined with the ship braking model, achieve a safer and more controllable ship braking process. By real-time monitoring and prediction of driver fatigue, appropriate measures can be taken in a timely manner to ensure navigation safety and driver health.

## 1. Introduction

Navigational safety has consistently been a primary concern in water transportation [1,2]. In particular, various complex environments such as strong winds and waves, channel reefs, congested waterways, and high current speeds can significantly impact ship braking and create considerable safety risks [3]. While intelligent driving technologies have been researched and applied to ship navigation, numerous challenges still need to be addressed in practical situations [4,5,6].

Investigating braking mechanisms for ship collision avoidance forms the foundation of navigational safety [7,8,9]. Goncharov et al. developed a ship braking process equation to address ship safety issues when navigating through ice channels. This equation served as a reference for modeling the braking process. Zhang et al. examined the characteristics of icebreakers in relation to the increased collision risk caused by a higher number of ships. This study proposed a model for determining safe braking distances for both escorting and escorted ships [10]. Yevgeniy et al. considered the inertial braking characteristics of ships and proposed a new calculation procedure for their inertial braking parameters, deriving expressions for both braking time and glide time. Using these expressions, their study established the relationship between the gliding distance and time when a ship brakes in the opposite direction, transitioning from full forward to full backward [11]. Burmaka et al. proposed a rapid collision risk avoidance method based on a ship’s braking pattern, subsequently developing a fast avoidance maneuver selection technique that actively changes a ship’s heading and speed through braking [8].

As the primary decision-maker in navigation, a pilot’s driving behavior and choices directly influence navigational safety [12]. Moreover, a ship pilot’s driving fatigue can also impact navigational safety. Driving fatigue plays a significant role in driving safety [13]. Driving fatigue will lead to longer reaction time of the pilot’s operation [14,15]. Numerous methods and techniques have been developed to address and enhance the examination of human factors in maritime navigational safety. Statistical investigations of navigation accident data are often limited to post-incident analyses [14]. Consequently, real-time collection and analysis of a ship pilot’s physiological data become increasingly crucial. Jiang et al. employed a 64-channel wireless EEG and EMG system, EEG signals, and EEGLAB to subjectively and objectively analyze and monitor driver fatigue levels [16]. Xiang et al. utilized a machine-learning approach to develop a driver fatigue recognition model based on bioelectrical signals. Yan et al. integrated oculomotor and subjective KSS scales with EEG data and driving environment information to develop a driving fatigue model based on multi-source data [17]. Hu et al. proposed a fatigue monitoring method based on EEG signals using information fuzzy entropy, the stability of which has been verified [18].

As intelligence and automation advance, research focusing on the human–ship–environment interaction is increasingly attracting scholarly attention [19]. The human–ship–environment interaction is crucial to understanding the impact of driver fatigue on navigational safety. Andrzej et al. develop a method to assess the effect of human factors on the risk of maneuvering accidents in restricted waters based on the human–ship–environment. The human factor encompasses crew members’ personality traits, stress tolerance, and risk avoidance. The developed human factor model enables targeted risk assessment for ships with crew members of varying characteristics, aiming to reduce traffic accident occurrences [20]. Wenke Zhao et al. subsequently investigated fuzzy mathematics and safety ergonomics principles, considering various factors such as navigational aids, water environment, ship equipment, and watchman quality. Their study introduced a proposed fuzzy mathematical model for evaluating ship driving fatigue [21].

This study investigates the impact of driver fatigue on ship braking safety by developing a human–ship–environment monitoring system and driver fatigue prediction model. The main contributions of this study include: (1) proposing a driver fatigue monitoring method based on the human–ship–environment system, (2) using the Stroop task experiment to induce driver fatigue response and selecting brainwave features related to fatigue severity scores, (3) constructing a ridge regression model to predict the driver’s fatigue level, (4) integrating the driver fatigue prediction model with the ship braking model to achieve a safer and more controllable ship braking process, and (5) monitoring and predicting driver fatigue in real-time to ensure timely implementation of appropriate measures for navigational safety and driver health. By analyzing the influence of driver fatigue on ship braking distance through a ship braking distance model and extracting features from EEG signals to establish a fatigue regression model, the feasibility of the human–ship–environment monitoring system to assist in emergency braking during hazardous navigation conditions is verified.

## 2. Materials and Methods

### 2.1. Data Acquisition

In this study, a total of 12 healthy participants, aged between 23 and 28 years, were recruited. All participants were non-smokers with no history of neurological or psychiatric disorders. They were instructed to maintain a normal sleep pattern and avoid alcohol or caffeine consumption within 24 h prior to the experiment.

The experiment was conducted in a quiet laboratory with a constant temperature of 24 °C. Participants were seated comfortably and wore an EEG cap. The EMOTIV device (14 channels, sampling rate of 128 Hz) was used to collect participants’ EEG signals.

For fatigue scoring, the Fatigue Severity Scale (FSS) was employed. The scale ranges from 0, indicating no fatigue or fatigue within the usual range, to 4, indicating extreme fatigue that hinders normal daily activities and work, requiring immediate rest and adjustment.

During data collection, participants were asked to perform the Stroop task to induce mental fatigue. The task involved displaying color words on a computer screen with the text color either consistent or inconsistent with the meaning of the word. Participants were required to respond to the text color by pressing the corresponding keys on the keyboard. Each participant’s experiment consisted of five sets with a one-minute break between sets. After each set, participants were required to rate their fatigue using the Fatigue Severity Scale.

### 2.2. Driving Intent Mechanisms

**Figure 1:**
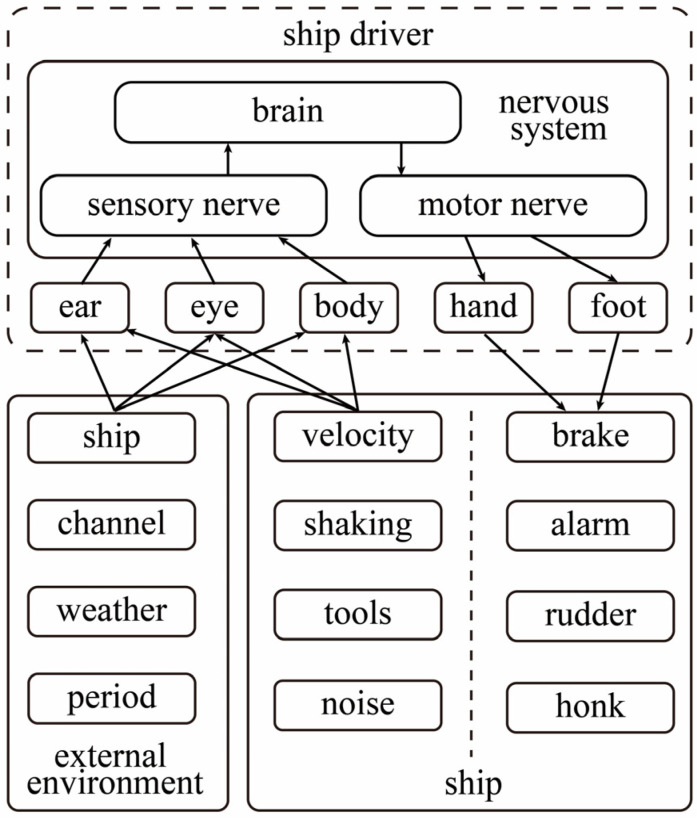
Driving behavior elements and their information interaction process.

Ship driving shares similarities with vehicle driving. Driving intention originates from the mental activity within the driver’s brain. Thus, to comprehend the conscious activities of the human brain, it is essential to initially investigate driving behavior. During driving, drivers receive a plethora of external information, including channel conditions, operations, and auditory information. Consequently, drivers must execute emergency response processing, relying on their personal experience, to alter their driving state [22]. Generally, drivers process the aforementioned information in a coherent manner, continuously adapting their state to meet the driving requirements. The execution of driving behavior is performed hierarchically over time by human functional organs. Thus, driving behavior can be defined as a generalization of diverse operating habits linked to different drivers, encompassing the perception, analysis, and processing of the external environment, as well as the driver’s internal operation of the ship [23]. Driving behavior integrates both internal and external factors. Figure 1 illustrates the driving behavior components and their corresponding information interaction process.

Driving intention, which originates from the brain without any observable manifestation, can only be analyzed through human physiological signals. Alternative methods are incapable of identifying and detecting a driver’s EEG information. Hence, to examine the EEG fatigue in a driver’s driving behavior, analysis should begin with the EEG information [24], focusing on driving behavior characteristics and the driver’s fatigue features. This approach can help mitigate potential hazards resulting from improper driving operations. To substantially decrease safety accidents resulting from incorrect operations and delayed driving behavior due to the driver’s intentions, the driver, ship, and environment must be considered holistically. Additionally, the features of a driver’s mental fatigue should be systematically identified and analyzed. Utilizing the drivers’ EEG signals, a ship-braking model, founded on the recognition of driving behavior consciousness, is developed.

### 2.3. Human–Ship–Environment System

**Figure 2:**
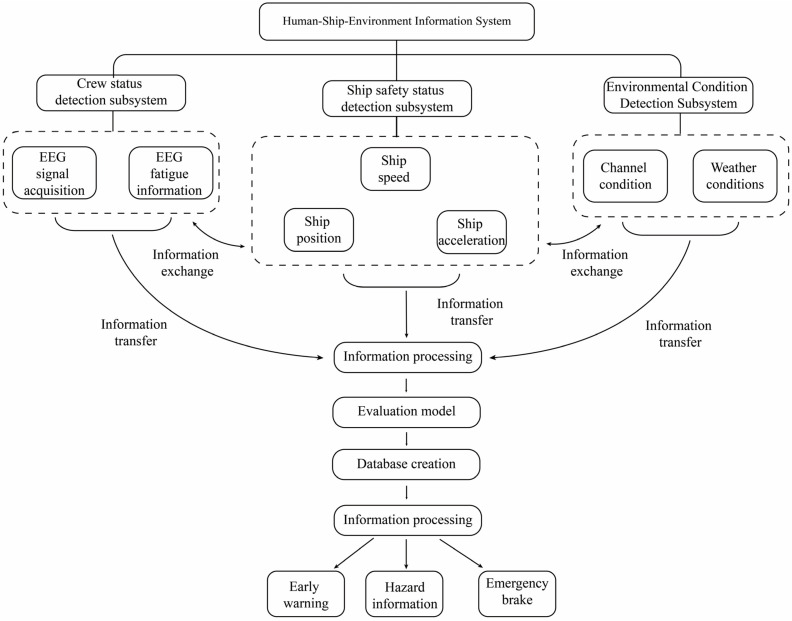
System functional architecture.

Navigation safety is influenced by the ship’s operating state, the driver’s physiological factors, and external environmental factors. A comprehensive consideration of these internal and external factors is necessary. Simultaneously, the fatigue state of the drivers, the sailing state of the ship, and the external environment should be monitored. As depicted in Figure 2, the functional architecture of the human–ship–environment system is as follows.

The human–ship–environment information system consists of three subsystems: the driver detection subsystem, the ship safety state detection subsystem, and the environmental state detection subsystem. Each subsystem is responsible for specific information collection and processing tasks. The driver status detection subsystem primarily monitors the driver’s condition, gathers real-time EEG signal information, and acquires mental fatigue data. The ship safety status detection subsystem focuses on monitoring real-time ship information, such as position changes, trajectory, and speed, to assess, among other aspects, navigation and braking safety. The environmental status detection subsystem primarily monitors external environmental conditions, including factors related to weather and water flow. When the equipment detects hazardous weather conditions, it issues a weather navigation warning. Simultaneously, the three subsystems facilitate information interaction and implement an integrated mechanism for human–ship–environment information.

Information gathered by the subsystem undergoes the information transmission process. Information processing tasks are executed within the information processing system. Information processing tasks encompass preprocessing, assessment using evaluation models, and data storage. Preprocessed information is assessed using the appropriate evaluation model. These data are stored in the designated database, ready for release by the information system as needed.

Once the information processing system has processed all data, any information deemed hazardous will be promptly sent to the information release system following evaluation. The driver will receive warnings as reminders. Primary information includes early warning details related to adverse weather, irregular ship operations, and other hazards.

**Figure 3:**
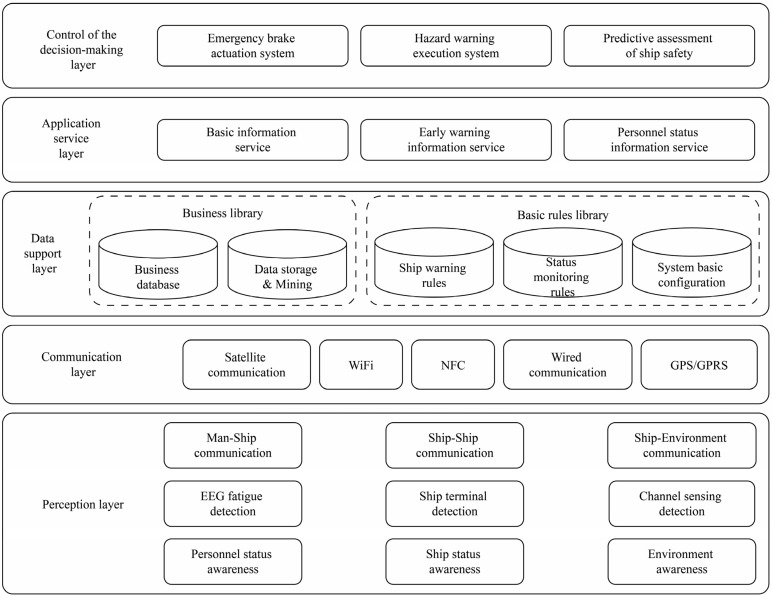
Technical architecture of personnel–ship–environmental information system.

As depicted in Figure 3, the overall design concept of the human–ship–environment monitoring system is centered on gathering human–ship–environment data. The system is designed utilizing a “practical and advanced” technological framework. Based on the principles of layering and separation, and the various functions of information technology, the system’s technical architecture model is derived and summarized by integrating the design outcomes of information and application architectures. This model primarily comprises the decision-making control layer, application service layer, data support layer, communication layer, and perception layer.

The perception layer handles the collection of the ship’s position, speed, and status information, driver status sensing and interaction, information data gathering and processing, as well as environmental data sensing, identification, and interaction. The communication layer, founded on information communication technology, facilitates information interaction between human, ship, and environment, encompassing information storage and feedback. The data support layer is split into a business library and a basic rule library. The business library stores human–ship–environment data. The basic rule library houses data pertaining to ship driving warning rules, status monitoring rules, and fundamental system configurations. The application service layer primarily offers personnel status detection services, ship operation status services, and waterway and environmental quality services to address the requirements of collaborative services involving personnel, ships, and the environment. The decision-making control layer compares pre-established target thresholds, conducting data analysis to derive the optimal control strategy. As a result, ship navigation hazards can be anticipated. Collaborative control of the human–ship–environment relationship can also be achieved.

A human–ship–environment system is developed based on functional and technical architectures. By monitoring multidimensional internal and external information, primarily centered on personnel brain fatigue, this system aids in expediting the emergency braking process, reducing the reaction time caused by driver fatigue, accelerating the ship’s emergency braking procedure, and decreasing the braking distance, ultimately enhancing the safety of the ship’s navigation.

### 2.4. Ship Breaking Model

**Figure 4:**
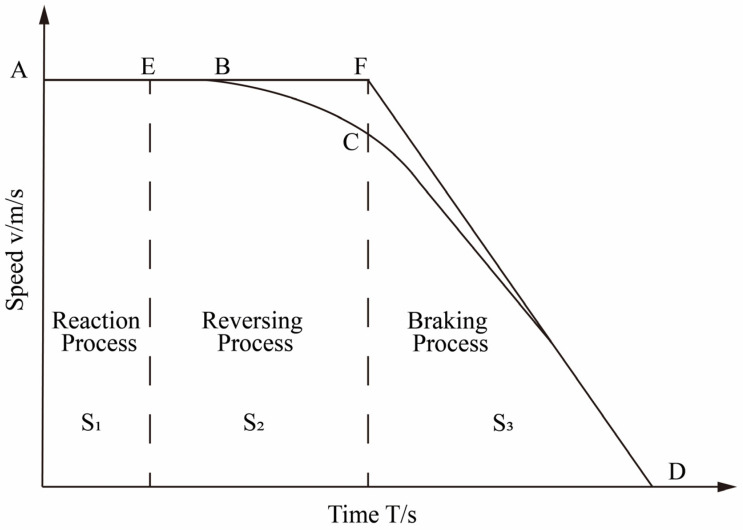
The relationship between braking speed and time.

The braking distance, which is closely related to shipping navigation safety, serves as a comprehensive performance evaluation index for braking. In general, the braking distance refers to the distance traveled after taking braking measures to stop. Figure 4 shows the relationship between speed and time in different stages of braking after receiving an emergency signal.

The braking process of a ship differs significantly from that of a car. Besides the operation reaction process of the driver, during the emergency braking process of the ship, the reverse propeller forms a reverse thrust to make the ship brake quickly. There is a reversing process before the ship enters the complete braking process. The engine stops at its normal speed and then reverses, reaching the maximum speed of reverse rotation.

Consequently, the ship’s emergency braking process primarily comprises three stages, manifested in the braking distance as the reaction stroke, reversing stroke, and braking stroke. The reaction stroke is the distance $S_{1}$ the ship travels at its original speed during the driver’s reaction time, which is related to the reaction speed of the ship’s driver. The reversing stroke is the distance $S_{2}$ that the ship moves during the reversing of the main engine. The braking stroke is the distance $S_{3}$ the ship moves while braking at full speed until it stops. The actual braking distance of the ship is ${S_{2}+S}_{3}$, while the safe braking distance encompasses $S_{1}+{S_{2}+S}_{3}$, spanning from the generation of the danger signal to the ship’s complete halt [25]. During the entire braking process, the speed of the ship is shown in the curve ABCD in Figure 4. In the reaction stage of the driver, the ship maintains the initial speed and moves forward at a constant speed within the reaction time $t_{1}$. After the driver performs the emergency braking operation, the engine starts to decelerate to stop and then accelerates at the reverse maximum speed, complete braking is entered at the reversing time $t_{2}$, and the ship’s deceleration reaches its maximum until the ship stops at $t_{3}$.

The forces acting on the ship during the reversing process are relatively complex. It is difficult to calculate the deceleration state of the ship and the reversing stroke. To simplify the calculation of the braking stroke during the ship’s braking process, the reversing process is considered as the ship moving forward at its initial speed. The braking process is that the ship decelerates at the maximum acceleration until the ship stops. As shown in Figure 4, The forces acting on the ship during the reversing process are complex, making it safer to use as the standard when predicting braking distance.

As per the simplified braking process AEFD presented in Figure 4, the braking distance, denoted as S, comprises the reaction process $S_{1}$, reversing process $S_{2}$, and braking process $S_{3}$.

(1) $$S=S_{1}+S_{2}+S_{3}$$

The simplified rear reaction process and reversing process (2) are as follows.

(2) $$\{\begin{array}{c} S_{1}=u\cdot t_{1}\\ S_{2}=u\cdot \left(t_{2}-t_{1}\right) \end{array}$$

The formula u is the speed of the ship. The driver’s reaction time $t_{1}$ is influenced by individual drivers and their ship-driving experience. Additionally, the driver’s fatigue state significantly impacts their reaction time. The reversing time ${(t}_{2}-t_{1})$ is related to the performance of different ships.

The braking process $S_{3}$ is closely related to the braking acceleration of the ship when it is sailing. The braking acceleration is affected by the power of the propeller motor and the water flow resistance of the ship. Therefore, the braking stroke of the ship is derived by the integral method as follows.

(3) $$S_{3}=\int_{0}^{u}\frac{mV^{2}}{RV+P}dV$$

In the formula, the integral V is the speed of the ship. m is the full-load displacement of the ship. R is the water flow resistance of the ship’s navigation. P is the maximum power of the ship’s propeller in reverse.

According to the Formulas (1)–(3), the braking distance of the ship can be obtained.

(4) $$S=u\cdot t_{1}+u\cdot \left(t_{2}-t_{1}\right)+\int_{0}^{u}\frac{mV^{2}}{RV+P}dV$$

It can be seen from Formula (4) that the main factors affecting the braking distance of the ship are the reaction time of the driver, the time of the ship’s reversing, and the braking performance parameters of the ship itself. According to the measured data of ship driving, after the ship’s speed reaches 20 km/h, the ship’s reversing time is within a certain range [26]. Therefore, the ship’s reversing time can be determined according to the ship’s model. The braking stroke is related to the ship’s model, full-load displacement, sailing power, and sailing water flow resistance. The resistance can be calculated according to JTS 144-1—2010 “Code for Port Engineering Loads”. Therefore, the braking stroke of the ship can be calculated to a certain value or within a certain range, which has a certain ratio relationship with the length of the ship. The calculated braking stroke of the ship closely resembles the measured braking stroke, serving as a reliable reference.

In the ship’s braking distance calculation, both the reversing stroke and braking stroke remain unaffected by human subjective factors. Given fixed objective conditions, such as navigation parameters, the controllable range of reversing stroke and braking stroke can be determined through calculations. The reaction stroke is affected by the driver. The reaction time has a large gap due to different personal subjective conditions. Particularly during emergency braking, the driver’s reaction time is profoundly influenced by their EEG-detected fatigue state. Hence, when a ship requires emergency braking under these conditions, assessing the impact of driver fatigue on reaction time becomes crucial.

## 3. Results and Discussion

### 3.1. EEG Fatigue Experiment

In studying the braking distance of ships, comprehensive and systematic monitoring and analysis of the ship’s operating state and external environment can lead to a more accurate assessment of the braking distance, thereby effectively ensuring navigation safety. Simultaneously, real-time monitoring of the driver’s fatigue state allows for prompt detection of the driver’s fatigue level, enabling the implementation of corresponding measures to guarantee the ship’s safety. Based on this, our study uses the Stroop task experiment to induce driver fatigue responses and investigate the relationship between driver fatigue characteristics and ship braking distance. Furthermore, we constructed a corresponding model to provide a scientific basis for the safe navigation of ships.

**Figure 5:**
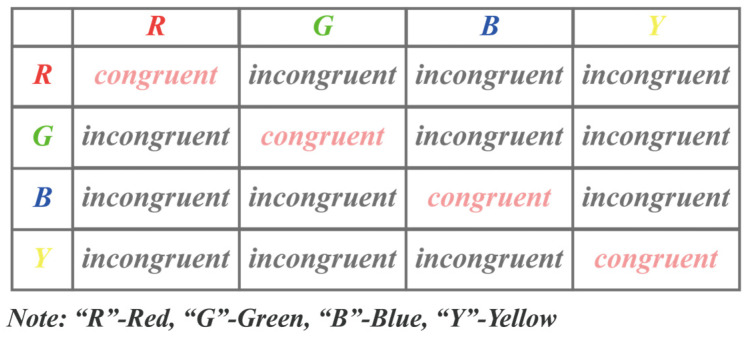
Sample composition of the Stroop task, generated by computer through 16 random combinations.

**Figure 6:**
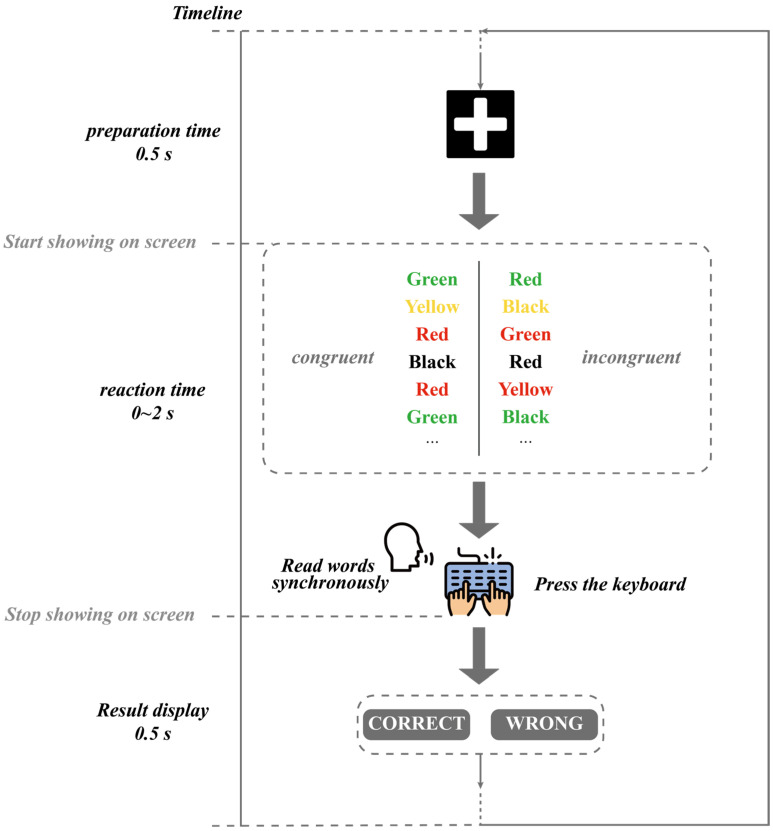
Flowchart of a single Stroop task trial.

**Figure 7:**
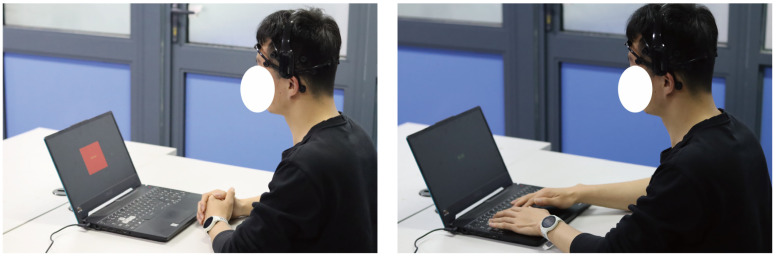
A participant wearing an EEG device during the Stroop task.

In this study, we utilized an online testing platform (https://www.psytoolkit.org/experiment-library/experiment_stroop.html, accessed on 17 April 2023) to conduct the Stroop task experiment, aiming to induce experimental fatigue in participants. Before conducting the experiment, ethical approval was obtained from the Shanghai University Institutional Review Board (IRB). All participants provided informed consent prior to participating in this study. The participants’ EEG signals were collected using the EMOTIV device, with a sampling rate of 128 Hz. The Stroop task has been widely utilized in research on fatigue induction [27]. As an attentional task, the Stroop task requires a high level of cognitive resources and attention control. Therefore, during the repeated execution of the Stroop task, participants may experience cognitive fatigue. In this study, the Stroop task consisted of 40 trials, generated randomly by the computer, and included 16 combinations of a word and the color it represents. Each combination presented either congruent or incongruent stimuli, as illustrated in Figure 5. Furthermore, Figure 6 shows the experimental procedure for inducing fatigue in participants using a single Stroop task. In each trial, a white cross is displayed on a black background by the computer to signal the start of the trial. After a 0.5 s delay, a random color will appear on the screen, and the participant needs to press one of the four keys on the keyboard, “R”, “G”, “B”, or “Y”, within 2 s to indicate the color of the displayed word. Each trial was recorded as one Stroop sample. During the measurement, participants were required to read the color of the presented word out loud. Each participant was required to complete five experimental trials, with a one-minute rest period between each trial. The participants were asked to complete the FSS Score to assess their level of fatigue. The detailed description of the FSS can be found in Table 1. A representative scene of a participant wearing an EMOTIV device while performing the Stroop task is depicted in Figure 7. This study aimed to investigate the relationship between fatigue levels and the power spectral characteristics of various EEG frequency bands by analyzing changes in the participants’ EEG signals.

### 3.2. Analysis of Stroop Data and Subjective Fatigue Scores

In the present study, the Stroop task was used to induce varying levels of fatigue. The experiment recorded participants’ response times and Stroop effect values under congruent and incongruent conditions. Five sets of data were collected for each participant, with the average response times and Stroop effect values for congruent and incongruent color conditions in the first and fifth sets being documented. These data were used to emphasize the impact of the Stroop task on the participants’ fatigue levels to the greatest extent possible. Additionally, subjective fatigue ratings were recorded, as presented in Table 2.

Based on the data presented in the table, it can be observed that the response times and Stroop effect values are generally higher under incongruent color conditions than those of congruent color conditions, suggesting that the Stroop task has a noticeable impact on the fatigue levels of the participants. Furthermore, the table also includes the participants’ subjective ratings of their fatigue, which can be used to validate the effectiveness of the Stroop task in inducing fatigue among the participants.

### 3.3. EEG Signal Preprocessing and Feature Extraction

Prior to analyzing the raw 14-channel data from the EMOTIV device, performing data cleaning and preprocessing is necessary. Preprocessing procedures include denoising, filtering, and equalization, among others. In this study, we utilized the EEGLAB toolbox for data processing. Denoising was carried out using Independent Component Analysis (ICA) by decomposing the data into independent components and removing noise components to minimize interference. Bandpass filtering was employed, with a cutoff frequency range of 1–30 Hz and a slope of 12 dB/octave. Equalization was achieved through normalization, processing the data for each channel to ensure equal potentials at the reference electrode. Finally, motion artifacts and faulty channels were removed from the preprocessed data to enhance signal quality and accuracy.

Subsequently, we employed PCA to reduce the dimensionality of the preprocessed EEG data, aiming to enhance the efficiency of feature extraction [28]. Through the PCA method, the 14-channel EEG data can be reduced to two principal components representing the most significant channels. From these two channels, two features CF and PSE were extracted [29,30]. CF reflects the concentration and migration trend of the majority of high-frequency components in the EEG signal, while PSE represents the complexity of the signal and the chaotic nature of its multi-frequency components. The formulas for these two features are provided below. Studies have demonstrated that these two features are closely associated with fatigue levels [30], prompting their selection as the most representative features for channel extraction in this study.

Following PCA analysis, channels 7 and 10 were chosen to represent the most characteristic channels among the 14 channels. The CF and PSE characteristics of channel 7 and channel 10 of the 12 subjects are shown in Table 3

(5) $$CF=\sum_{i=1}^{n}f_{i}\cdot P\left(f_{i}\right)/\sum_{i=1}^{n}P\left(f_{i}\right)$$

where $f_{i}$ denotes frequency and, ${P(f}_{i})$ represents the power spectral density at the given frequency.

The formula for calculating power spectral entropy is as follows:

(6) $$PSE=-\sum_{i=1}^{n}P\left(f_{i}\right)\cdot \log_{2}\left(P\left(f_{i}\right)\right)$$

where ${P(f}_{i})$ also represents the power spectral density at the given frequency.

### 3.4. Correlation Analysis of CF/PSE

**Figure 8:**
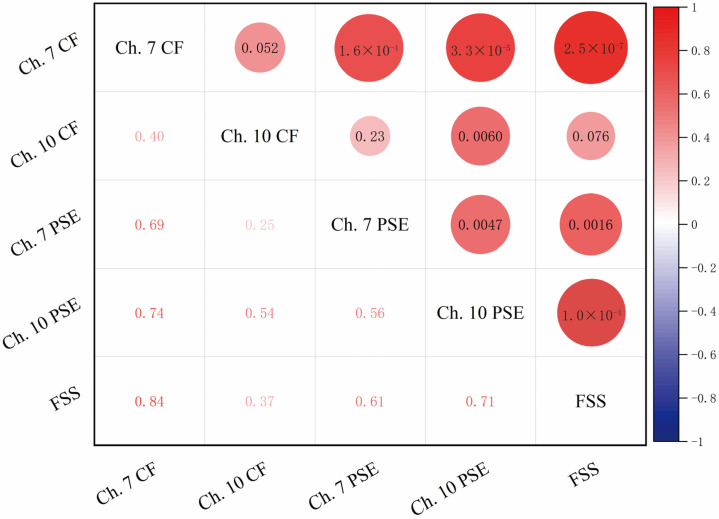
Correlation analysis of CF/PSE of channel 7 and 10 with FSS (lower-left half represents correlation coefficients, upper-right half represents *p*-values).

As illustrated in Figure 8 and based on the data from the table, we conducted a correlation analysis. This study found a highly significant positive correlation between CF of channel 7 and FSS subjective fatigue scores (r = 0.84, *p* = 2.5 × 10^−7^ < 0.05). This suggests that when the CF value of channel 7 is higher, participants’ subjective fatigue scores are also higher, indicating that the CF of channel 7 can effectively reflect the fatigue levels of the participants. Moreover, a significant positive correlation was observed between the PSE of channel 7 and the FSS Score (r = 0.61, *p* = 0.0016 < 0.05), indicating that when the PSE value of channel 7 is higher, the subjective fatigue scores of participants also increase. This suggests that the PSE value of channel 7 can serve as another indicator of the participants’ subjective fatigue levels. In comparison with channel 7, the correlation between the CF of channel 10 and the FSS subjective fatigue scores is weaker. Although it still exhibits a positive trend (r = 0.37, *p* = 0.076 > 0.05), it does not reach a significant level. The correlation between the PSE of channel 10 and the FSS Score is also relatively weak, displaying a positive trend (r = 0.71, *p* = 1.0 × 10^−4^ < 0.05), but it does not reach the highly significant level as observed with the PSE of channel 7. However, the significance indicators are within a reasonable range and can be used collectively for constructing a regression model for fatigue levels.

### 3.5. Fatigue Model Construction Based on Ridge Regression

In this study, based on the correlation analysis results presented earlier, a ridge regression model was constructed using the CF and PSE features of channels 7 and 10 to predict participants’ fatigue scores. Channel 7’s CF and PSE, as well as channel 10’s PSE, were selected as input parameters for the model. This selection was made due to the findings from the correlation analysis, which revealed that channel 7’s CF and PSE had a highly significant positive correlation with the FSS fatigue scores, whereas the correlation between channel 10’s CF and the FSS Score was relatively weak and did not reach significance. Consequently, the three features—CF and PSE from channel 7, and PSE from channel 10—were chosen as the most representative channels for feature extraction and were employed to construct the regression model for fatigue scores.

To construct the ridge regression model, an appropriate regularization parameter α is required to balance the model’s complexity and fitting performance. The optimal α value can be selected through cross-validation, which achieves the minimum mean squared error on the validation set. The objective of ridge regression is to find a coefficient vector β that minimizes the ridge regression loss function, as shown below:

(7) $$\min_{\beta }\left\{\sum_{i=1}^{n}{\left(y_{i}-\beta_{0}-\sum_{j=1}^{p}\beta_{j}x_{ij}\right)}^{2}+\alpha \sum_{j=1}^{p}\beta_{j}^{2}\right\}$$

In Equation (7), $\beta_{0}$ is the intercept, $\beta_{1},\beta_{2},\ldots ,\beta_{p}$ are the feature coefficients, and α is the regularization parameter. When α=0, ridge regression degenerates into least squares regression. As α approaches infinity, all feature coefficients tend towards 0, simplifying the model. The coefficient vector β can be obtained by solving the following system of equations:

(8) $$\left(X^{T}X+\alpha I\right)\beta =X^{T}Y$$

In Equation (8), I is the identity matrix. Since the sample size in this study is relatively small, an analytical solution is used to solve for the coefficient vector β.

Ultimately, the fatigue evaluation value $\hat{y}$ of the ridge regression model can be expressed as follows:

(9) $$\hat{y}=\beta_{0}+\sum_{j=1}^{p}\beta_{j}x_{j}$$

where $x_{j}$ is the j feature value in the test sample.

In this study, CF and PSE of channel 7 and PSE of channel 10 were used as input variables, with FSS subjective fatigue scores as the output variable. The ridge regression method was employed for model training and testing. The optimal regularization parameter α was determined to be 0.1 through cross-validation. The final ridge regression model can be expressed as follows:

(10) $$FSS=0.86CF_{ch7}+0.06PSE_{ch7}+0.15PSE_{ch10}-0.04$$

In Equation (10), ${CF}_{ch7}$ denotes the CF value of channel 7, ${PSE}_{ch7}$ denotes the PSE value of channel 7, ${PSE}_{ch10}$ denotes the PSE value of channel 10. The coefficient −0.04 indicates that there is some collinearity among these three features, which necessitates the introduction of a regularization term to avoid overfitting.

In this study, a ridge regression model was constructed to evaluate its predictive capability and stability, utilizing a five-fold cross-validation method. Specifically, the dataset was divided into five parts, with four parts serving as the training set and one part as the validation set for model training and validation. The average validation error was obtained by averaging the results of the five validations.

**Figure 9:**
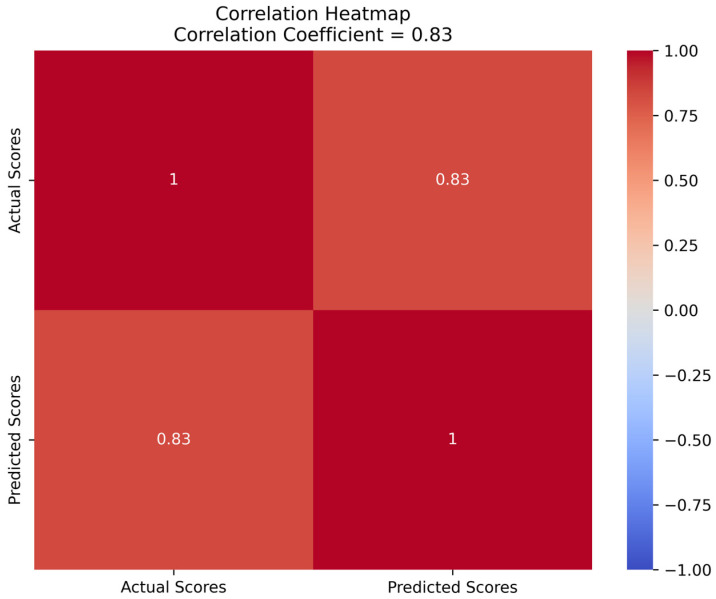
Correlation heatmap of predicted and actual fatigue scores.

Through the five-fold cross-validation, the average validation error of the ridge regression model was found to be 0.36, indicating good predictive ability. To further evaluate the model’s performance, a correlation heatmap (Figure 9) was generated, illustrating the strength of the linear relationship between the predicted and actual fatigue scores. The correlation coefficient between the predicted and actual values was 0.83, demonstrating a strong linear relationship between the model’s predictions and the actual values. These results suggest that the ridge regression model can effectively predict the subjective fatigue scores of the participants and possesses high stability and generalization capability.

### 3.6. Discussion

In this study, a human–ship–environment monitoring system for ships was developed, enabling the monitoring of driver fatigue levels and its integration with ship braking models to enhance braking safety. For driver fatigue monitoring, the Stroop task experiment was utilized to induce driver fatigue response. The CF and PSE features from channels 7 and 10 were selected and then subjected to a correlation analysis with the FSS Score. Subsequently, a ridge regression model was constructed to predict the driver’s fatigue level. This model assists in real-time detection of driver fatigue during ship navigation, allowing targeted measures to be taken to improve ship braking safety.

In comparison to previous studies, our ridge regression model offers unique contributions to the field of driver fatigue detection based on EEG features. Unlike the study by AlShorman et al. [31], which focuses on mental stress detection using machine-learning techniques and EEG analysis of the frontal lobe, our study specifically targets driver fatigue in the context of a human–ship–environment monitoring system. In addition, our research differs from the work of Bin Heyat et al. [32], which employs wearable flexible electronics and machine-learning models for mental stress detection based on single-lead ECG signals. Our study, on the other hand, concentrates on enhancing ship braking safety through driver fatigue monitoring.

Furthermore, our work is distinct from the study by Bin Heyat et al. [33], which investigates the role of oxidative stress and inflammation in insomnia sleep disorders and cardiovascular diseases, and proposes a machine-learning-based insomnia detection system using ECG-derived R–R intervals. Our ridge regression model, by contrast, aims to predict driver fatigue levels with high stability and generalization capability.

However, certain limitations exist in this study. Firstly, the limited sample size may affect the accuracy and stability of the predictive model. To resolve this issue, future research could involve expanding the sample size to improve the model’s performance. Secondly, this study focused on only a few EEG features; future research could analyze driver fatigue characteristics from multiple perspectives, such as incorporating additional physiological signals or machine-learning techniques, to optimize the predictive model. Additionally, driver fatigue may be influenced by various factors, such as driving duration and work pressure. To further enhance the comprehensiveness of fatigue prediction, future research could consider these factors and develop more personalized fatigue management strategies.

## 4. Conclusions

This study aims to address the uncontrolled risks posed by the excessive reliance on crew driving experience in existing ship safety braking methods. To this end, our study focuses on reducing the impact of driver fatigue on ship driving safety, initially constructing a functional and technical architecture of a human–ship–environment monitoring system for ships. Furthermore, a ship braking model considering personnel brain fatigue monitoring was investigated to reduce braking safety risks during ship navigation.

To better investigate the impact of driver fatigue on ship braking safety, this study designed a Stroop task experiment. The purpose of this experiment was to induce fatigue in participants simulating ship drivers. During the experiment, participants were required to complete various cognitive tasks while fatigued, allowing for the investigation of their responses and performance under fatigue conditions. Through this approach, a better understanding of the relationship between the behavior of ship drivers in a fatigued state and ship braking safety can be gained.

By utilizing the Stroop task experiment to induce driver fatigue response, in this study we selected the CF and PSE features from channels 7 and 10 and conducted a correlation analysis with the fatigue FSS Score. A ridge regression model was constructed to predict the driver’s fatigue level, providing a scientific basis for real-time monitoring of driver fatigue. Through real-time monitoring and prediction of driver fatigue, appropriate measures can be taken in a timely manner to ensure navigational safety and driver health.

To further enhance the impact of this study, future research directions include expanding the sample size to improve the performance and stability of the predictive model, analyzing driver fatigue characteristics from multiple perspectives to optimize the predictive model, and considering factors such as driving duration and work pressure to enhance the comprehensiveness of fatigue prediction.

In conclusion, this study has developed a human-ship-environment monitoring system by utilizing a Stroop task experiment, which helps understand the relationship between fatigued ship drivers and ship braking safety. A driver fatigue prediction model is inte-grated with ship braking models, providing real-time monitoring and prediction of driver fatigue, ensuring navigational safety, and driver health. This research has significant im-plications for improving ship braking safety and offers protection for navigational safety and driver health.

## Figures and Tables

**Table 1:** The 5-point Fatigue Severity Scale.

Score	Description
0	No fatigue or fatigue within the usual range experienced by the individual
1	Slightly or briefly above the individual’s typical fatigue level
2	Noticeable fatigue, requiring some effort to cope with
3	Significant fatigue, affecting daily activities and work performance
4	Extreme fatigue, rendering the individual incapable of performing normal daily activities and work, necessitating immediate rest and recovery

**Table 2:** Distribution of response times, Stroop effect values, and subjective fatigue scores for the 12 participants in the Stroop task.

Sub ID\Condition	Average Color Congruent Time (ms)	Average Color Incongruent Time (ms)	Stroop Effect Value (ms)	FSS Score
1	790	1118	328	0.5
	856	1042	186	1.5
2	978	1245	267	0
	655	923	268	2
3	921	1154	233	0
	1062	1229	167	1
4 *	958	898	−60	0.5
	894	830	−64	0.5
5	898	1100	202	0.5
	1235	1449	214	2
6	729	999	270	1
	1098	1378	280	2.5
7	872	1023	151	0
	1036	1330	294	1.5
8	837	1025	188	0.5
	1267	1543	276	2.5
9	945	1201	256	1
	963	1124	161	1.5
10	991	1042	51	0.5
	731	1079	348	3
11	936	1071	135	0
	1022	1215	193	1.5
12	720	1011	291	0.5
	858	1173	315	2.5

* Refers to the fifth set of data for participant 4, where the average response time for congruent color conditions was higher than that for incongruent color conditions, resulting in a negative Stroop effect value. This data point was excluded from the model construction.

**Table 3:** CF and PSE feature data of channels 7 and 10 in 12 subjects.

Subject No.	CF of Channel 7	CF of Channel 10	PSE of Channel 7	PSE of Channel 10
1	6.7	7.1	0.72	0.68
	7.2	7.4	0.86	0.79
2	6.5	6.8	0.78	0.71
	7.9	7.5	0.89	0.77
3	6.1	5.8	0.62	0.58
	7.3	5.9	0.67	0.72
4 *	7.8	7.3	0.81	0.77
	6.8	7.2	0.73	0.76
5	6.2	6.9	0.65	0.62
	7.6	7.5	0.79	0.78
6	7.1	7.1	0.75	0.72
	7.6	7.5	0.86	0.93
7	5.6	6.8	0.61	0.61
	7.3	7.3	0.82	0.73
8	6.8	7.2	0.77	0.69
	8	7.4	0.83	0.75
9	7.1	7.3	0.73	0.76
	7.8	8.2	0.81	0.88
10	6.5	7.5	0.7	0.81
	8.6	7.8	0.95	0.84
11	7.2	7	0.86	0.69
	7.7	7.7	0.75	0.83
12	6.7	7.4	0.72	0.78
	8.7	7.1	0.72	0.88

The * number in Table 3 is subject No. 4. Since the value of the Stroop effect in the Stroop experiment is negative, it was discarded in the model construction.

## Data Availability

All data included in this study are available upon request by contact with the corresponding author. The data are not publicly available because of ethical restrictions.
